# The Lattices of Group Fuzzy Congruences and Normal Fuzzy Subsemigroups on *E*-Inversive Semigroups

**DOI:** 10.1155/2014/413564

**Published:** 2014-05-05

**Authors:** Shoufeng Wang

**Affiliations:** Department of Mathematics, Yunnan Normal University, Kunming, Yunnan 650500, China

## Abstract

The aim of this paper is to investigate the lattices of group fuzzy congruences and normal fuzzy subsemigroups on *E*-inversive semigroups. We prove that group fuzzy congruences and normal fuzzy subsemigroups determined each other in *E*-inversive semigroups. Moreover, we show that the set of group *t*-fuzzy congruences and the set of normal subsemigroups with tip *t* in a given *E*-inversive semigroup form two mutually isomorphic modular lattices for every *t∈* [0,1].

## 1. Introduction

The investigation of fuzzy sets is initiated by Zadeh in [1]. As special fuzzy sets, fuzzy congruences on groups and semigroups have been extensively studied by many authors. In 1992, Kuroki [2] introduced fuzzy congruences on a group and characterized fuzzy congruences on a group in terms of fuzzy normal subgroups. In 1993, Samhan [3] studied the modularity condition in the fuzzy congruence lattice of a semigroup and derived that the fuzzy congruence lattice of a group is modular. In the same year, Al-Thukair [4] described the fuzzy congruences of an inverse semigroup and obtained a one-one correspondence between fuzzy congruence pairs and fuzzy congruences on an inverse semigroup. Moreover, Kuroki also studied the fuzzy congruences on inverse semigroups in [5] in which the notion of group congruences of a semigroup is provided. Das [6] considered the lattice of fuzzy congruences in an inverse semigroup by kernel-trace approaches. In 1995, Ajmal and Thomas considered the lattice structures of fuzzy congruences on a group and the lattice structures of fuzzy subgroups and fuzzy normal subgroups in a group in [7] and proved that the lattice of fuzzy normal subgroups of a group is modular in [8]. In 1997, Kim and Bae [9] studied the fuzzy congruences of groups and obtained several results which are analogs of some basic theorems of group theory. Also, Xie [10] studied the so-called fuzzy Rees congruences on semigroups in 1999.

Several authors investigated fuzzy congruences for some special classes of semigroups. In 2000, Zhang [11] characterized the group fuzzy congruences on a regular semigroup by some fuzzy subsemigroups. In 2001, Tan [12] investigated fuzzy congruences of regular semigroups and proved that the lattice of fuzzy congruences on a regular semigroup is a disjoint union of some modular sublattices of the lattice. Recently, Li and Liu [13] characterized fuzzy good congruences of left semiperfect abundant semigroups and obtained sufficient and necessary conditions for an abundant semigroup to be left semiperfect.

The class of *E*-inversive semigroups is a very wide class of semigroups which contains groups, inverse semigroups, and regular semigroups as proper subclasses and some kinds of crisp congruences on this class of semigroups have been investigated extensively; see [14, 15] for example. In particular, Gigoń [14] considered the lattice of group crisp congruences on an *E*-inversive semigroup and proved that this lattice is modular. Inspired by the above facts, it is natural to study the fuzzy congruences on *E*-inversive semigroups. In fact, [16] has done some works in this aspect.

In this paper, we shall investigate the lattices of group fuzzy congruences and normal fuzzy subsemigroups on an *E*-inversive semigroup. The notions of group *t*-fuzzy congruences and normal fuzzy subsemigroups with tip *t* on *E*-inversive semigroups are proposed and some properties of them are given. In particular, for a given *E*-inversive semigroup *S*, we prove that for any *t* ∈ [0,1] the set of group *t*-fuzzy congruences and the set of normal fuzzy subsemigroups with tip *t* on *S* form two mutually isomorphic modular lattices. Our results generalize and enrich several results obtained in [2, 3, 8, 9, 11, 14]. Notations and terminologies not given in this paper can be found in [17–19].

## 2. Preliminaries

A binary relation “≤” defined on a set *A* is a* partial order* on the set *A* if the following conditions hold identically in *A*:

(1)  *a* ≤ *a*; (2)  *a* ≤ *b* and *b* ≤ *a* imply *a* = *b*; (3)  *a* ≤ *b* and *b* ≤ *c* imply *a* ≤ *c*.

Let *A* be a subset of a poset (*P*, ≤). An element *p* in *P* is an upper bound for *A* if *a* ≤ *p* for every *a* in *A*. An element *p* in *P* is the* supremum* of *A* if *p* is an upper bound of *A* and *p* is the smallest among the upper bounds of *A*. Dually, we can define the* infimum* of *A*. We denote the supremum and the infimum of *A* by ⋁_*a*∈*A*_ *a* and ⋀_*a*∈*A*_ *a*, respectively.

A poset (*L*, ≤) is called a* lattice* if for every *a*, *b* in *L* both the supremum *a*∨*b* and the infimum *a*∧*b* of {*a*, *b*} exist in *L*. A* modular lattice* is any lattice *L* which satisfies the* modular law*: *x* ≤ *y* implies that *x*∨(*y*∧*z*) = *y*∧(*x*∨*z*) for all *x*, *y*, *z* in *L*.

Two lattices *L*_1_ and *L*_2_ are* isomorphic* if there is a bijection *α* from *L*_1_ to *L*_2_ such that for every *a*, *b* in *L*_1_ the following two equations hold: *α*(*a*∨*b*) = *α*(*a*)∨*α*(*b*) and *α*(*a*∧*b*) = *α*(*a*)∧*α*(*b*). If *P*_1_ and *P*_2_ are two posets and *α* is a map from *P*_1_ to *P*_2_, then we say *α* is* order-preserving* if *α*(*a*) ≤ *α*(*b*) holds in *P*_2_ whenever *a* ≤ *b* holds in *P*_1_.

On the theory of lattices, we need the following results.

**Lemma 1 (Theorem 2.3 of Chapter 1 in [17])**

Two lattices *L*_1_ and *L*_2_ are isomorphic if and only if there is a bijection *α* from *L*_1_ to *L*_2_ such that both *α* and *α* ^−1^ are order-preserving.

**Lemma 2 (Theorem 4.2 of Chapter 1 in [17])**

Let *P* be a poset such that it has the largest element and the infimum of every nonempty subset exists. Then *P* is a lattice.

Zadeh [1] defined a fuzzy subset *μ* in a set *S* as a mapping from *S* to the closed unit interval [0,1]. A fuzzy set *μ* in a set *S* is said to be contained in a fuzzy set *η* if *μ*(*x*) ≤ *η*(*x*) for all *x* in *S* and this is denoted by *μ*⊆*η*. The union *μ* ∪ *η* and the intersection *μ*∩*η* of two fuzzy sets *μ* and *η* in a set *S* are defined by

(1) $$\begin{array}{c} \mu \cup \eta \left(x\right)=\max\left(\mu \left(x\right),\eta \left(x\right)\right)=\mu \left(x\right)\vee \eta \left(x\right),\\ \mu \cap \eta \left(x\right)=\min\left(\mu \left(x\right),\eta \left(x\right)\right)=\mu \left(x\right)\wedge \eta \left(x\right) \end{array}$$

for all *x* in *S*. Further, if *μ*_*i*_ is a fuzzy subset in *S* for *i* ∈ *I* where *I* is an index set, then ⋂_*i*∈*I*_ *μ*_*i*_ is defined by

(2) $$\begin{array}{c} \underset{i\in I}{⋂}\mu_{i}\left(x\right)=\inf\left\{\mu_{i}\left(x\right)\;|\;i\in I\right\}\;=\underset{i\in I}{⋀}\mu_{i}\left(x\right) \end{array}$$

for all *x* ∈ *S*.

A* semigroup* is a nonempty set with an associative binary operation. A semigroup *S* is called* E-inversive* if for all *a* ∈ *S* there exists *a*′ ∈ *S* such that *a*′*aa*′ = *a*. In this case, we denote *W*(*a*) = {*a*′ ∈ *S* | *a*′*aa*′ = *a*′} and call the elements in *W*(*a*) the* weak inverses* of *a* for any *a* ∈ *S*. It is easy to see that groups, regular semigroups, and semigroups with zeros are all *E*-inversive semigroups. For more details on *E*-inversive semigroups, see [14, 15] and their references. Throughout this paper, we always assume that *S* is an *E*-inversive semigroup and let

(3) $$\begin{array}{c} E\left(S\right)=\left\{e\in S\;|\;e^{2}=e\right\}. \end{array}$$

Now, we give the concept of *t*-fuzzy congruences.

**Definition 3 (see [19])**

Let *t* ∈ [0,1]. A *t*-fuzzy equivalence on *S* is a fuzzy subset in *S* × *S* which satisfies the following conditions:

1. (∀*a* ∈ *S*)  *ρ*(*a*, *a*) = *t*,
2. (∀*a*, *b* ∈ *S*)  *ρ*(*a*, *b*) = *ρ*(*b*, *a*) ≤ *t*,
3. (∀*a*, *b*, *c* ∈ *S*)  *ρ*(*a*, *b*) ≥ *ρ*(*a*, *c*)∧*ρ*(*c*, *b*).

A *t*-fuzzy equivalence *ρ* on *S* is called a *t*-*fuzzy* *congruence* if

(4) (∀*a*, *b*, *c* ∈ *S*)  *ρ*(*a*, *b*) ≤ *ρ*(*ac*, *bc*)∧*ρ*(*ca*, *cb*),

or, equivalently,

(4′) (∀*a*, *b*, *c*, *d* ∈ *S*)  *ρ*(*ab*, *cd*) ≥ *ρ*(*a*, *c*)∧*ρ*(*b*, *d*).

Similar to the proofs of Lemmas 2.2 and 2.3 in Kuroki [5], we have the following results.

**Lemma 4**

Let *ρ* be a *t*-fuzzy congruence on *S*. For any *a* ∈ *S*, define a fuzzy subset *ρ*_*a*_ in *S* as follows: *ρ*_*a*_(*x*) = *ρ*(*a*, *x*) for all *x* ∈ *S*.

1. *ρ*_*a*_ = *ρ*_*b*_ if and only if *ρ*(*a*, *b*) = *t* for all *a*, *b* ∈ *S*.
2. *S*/*ρ* = {*ρ*_*a*_ | *a* ∈ *S*} is a semigroup with the multiplication *ρ*_*a*_ *ρ*_*b*_ = *ρ*_*ab*_ for any *a*, *b* ∈ *S*.

## 3. Group Fuzzy Congruences

In this section, we consider some basic properties of group fuzzy congruences on *S*. In particular, we show that the set of group *t*-fuzzy congruences on *S* forms a modular lattice. Firstly, we give the concept of group *t*-fuzzy congruences which is parallel to that of usual group fuzzy congruences defined in Kuroki [5].

**Definition 5**

A *t*-fuzzy congruence *ρ* on *S* is called a group *t*-*fuzzy  congruence* if the semigroup *S*/*ρ* is a group. One denotes the set of group *t*-fuzzy congruences on *S* by **G** **F** **C**_*t*_(*S*) and denotes **G** **F** **C**(*S*) = ⋃_*t*∈[0,1]_ **G** **F** **C**_*t*_(*S*).

The following result provides a characterization of group *t*-fuzzy congruences on *S*.

**Proposition**

A *t*-fuzzy congruence *ρ* on *S* is a group *t*-fuzzy congruence if and only if

1. (∀*e*, *f* ∈ *E*(*S*))  *ρ*(*e*, *f*) = *t;*
2. (∀*a* ∈ *S*)(∀*a*′ ∈ *W*(*a*))  *ρ*(*aa*′*a*, *a*) = *t*.

**Proof**

If *ρ* is a group *t*-fuzzy congruence, then *S*/*ρ* is a group and so *ρ*_*e*_ is the identity of *S*/*ρ* for every *e* ∈ *E*(*S*). This implies that *ρ*(*e*, *f*) = *t* for all *e*, *f* ∈ *E*(*S*) by Lemma 4. Furthermore, since *a*′*a* ∈ *E*(*S*) for any *a* ∈ *S* and *a*′ ∈ *W*(*a*), it follows that *ρ*_*a*′*a*_ is the identity of *S*/*ρ* and so *ρ*_*a*_ = *ρ*_*a*_ *ρ*_*a*′*a*_ = *ρ*_*aa*′*a*_. This yields that *ρ*(*aa*′*a*, *a*) = *t* by Lemma 4 again.

Conversely, let *a* ∈ *S*, *a*′ ∈ *W*(*a*), and *e* ∈ *E*(*S*). Then by condition (1) and Lemma 4, *ρ*_*aa*′_ = *ρ*_*a*′*a*_ = *ρ*_*e*_. By condition (2) and Lemma 4, we have

(4) $$\begin{array}{c} \rho_{a}=\rho_{aa^{\prime }a}=\rho_{a}\rho_{a^{\prime }a}=\rho_{a}\rho_{e},\;\;\;\rho_{a}\rho_{a^{\prime }}=\rho_{aa^{\prime }}=\rho_{e}, \end{array}$$

which implies that *S*/*ρ* is a group.

**Proposition 7**

Let *ρ* ∈ **G** **F** **C**_*t*_(*S*), *a*, *b* ∈ *S*, and *e* ∈ *E*(*S*). Then

(5) $$\begin{array}{c} \rho \left(a,b\right)=\rho \left(a,be\right)=\rho \left(ae,b\right)=\rho \left(a,eb\right)=\rho \left(ea,b\right). \end{array}$$

**Proof**

Since *ρ* ∈ **G** **F** **C**_*t*_(*S*), by Proposition 6, we have

(6) $$\begin{array}{c} \rho \left(b,bb^{\prime }b\right)=\rho \left(a,aa^{\prime }a\right)=t=\rho \left(aa^{\prime },e\right)=\rho \left(e,b^{\prime }b\right) \end{array}$$

for all *a*′ ∈ *W*(*a*) and *b*′ ∈ *W*(*b*). This implies that

(7) ρ(a,be)≥ρ(a,aa′a)∧ρ(aa′a,be)=ρ(aa′a,be)≥ρ(a,b)∧ρ(aa′,e)=ρ(a,b).

On the other hand,

(8) ρ(a,b)≥ρ(a,be)∧ρ(be,bb′b)∧ρ(bb′b,b)≥ρ(a,be)∧ρ(be,bb′b)≥ρ(a,be)∧ρ(b,b)∧ρ(e,b′b)=ρ(a,be).

Therefore, *ρ*(*a*, *b*) = *ρ*(*a*, *be*). By similar arguments, we can show

(9) $$\begin{array}{c} \rho \left(a,b\right)=\rho \left(ae,b\right)=\rho \left(a,eb\right)=\rho \left(ea,b\right) \end{array}$$

for all *a*, *b* ∈ *S* and *e* ∈ *E*(*S*).

As usual, for *σ*, *τ* ∈ **G** **F** **C**_*t*_(*S*), we define *σ*∘*τ* as follows:

(10) $$\begin{array}{c} \sigma \circ \tau \left(a,b\right)=\underset{x\in S}{⋁}\left(\sigma \left(a,x\right)\wedge \tau \left(x,b\right)\right) \end{array}$$

for all *a*, *b* ∈ *S*. Then we have the following.

**Proposition**

Let *σ*, *τ* ∈ **G** **F** **C**_*t*_(*S*).

1. *σ*∘*τ* = *τ*∘*σ*.
2. *σ*∘*τ is the least group t-fuzzy congruence of  S containing σ and τ*.
3. *σ*∩*τ is the greatest group t-fuzzy congruence of S contained in σ and τ*.

**Proof**

(1) For all *a*, *b* ∈ *S* and *c*′ ∈ *W*(*c*), by Proposition 7, we have

(11) σ∘τ(a,b)=⋁x∈S(σ(a,x)∧τ(x,b))≥⋁c∈S(σ(a,bc′a)∧τ(bc′a,b))=⋁c∈S(τ(bc′a,b)∧σ(a,bc′a))=⋁c∈S(τ(bc′a,bc′c)∧σ(cc′a,bc′a))≥⋁c∈S(τ(a,c)∧σ(c,b))=τ∘σ(a,b).

By symmetry, we have *σ*∘*τ*(*a*, *b*) = *τ*∘*σ*(*a*, *b*) for all *a*, *b* ∈ *S*.

(2) Let *a*, *b*, *c* ∈ *S*. Since

(12) t=σ(a,a)∧τ(a,a)≤⋁x∈S(σ(a,x)∧τ(x,a))≤⋁x∈S(t∧t)=t,

we have *σ*∘*τ*(*a*, *a*) = *t*. Similarly, we have

(13) σ∘τ(a,b)=⋁x∈S(σ(a,x)∧τ(x,b))≤⋁x∈S(t∧t)=t.

In view of the proofs of Propositions 1.8 and 1.9 in Kim and Bae [9], *σ*∘*τ* is the least *t*-fuzzy congruence on *S* containing *σ* and *τ*. Finally, we can easily show that *σ*∘*τ* ∈ **G** **F** **C**_*t*_(*S*) by Proposition 6. This implies that *σ*∘*τ* is the least group *t*-fuzzy congruence of *S* containing *σ* and *τ*.

(3) This is clear.

By Proposition 8 and the proof of Theorem 1.12 in Kim and Bae [9], we have the following result.

**Theorem 9**

(**G** **F** **C**_*t*_(*S*), ⊆) forms a modular lattice for any *t* in [0,1].

In the end of this section, we give some properties of group *t*-fuzzy congruences on *S* which will be used in the final section.

**Proposition**

Let *ρ* ∈ **G** **F** **C**_*t*_(*S*) and *a*, *b* ∈ *S*.

1. *ρ*(*aa*′, *a*) = *ρ*(aa*, *a*)* for all a*′, a* ∈ *W*(*a*).
2. *ρ*(*a*′*b*(*a*′*b*)′, *a*′*b*) = *ρ*(*a*, *b*)* for all a*′ ∈ *W*(*a*)* and *(*a*′*b*)′ ∈ *W*(*a*′*b*).

**Proof**

(1) Since *ρ* ∈ **G** **F** **C**_*t*_(*S*), by Proposition 6, we have

(14) $$\begin{array}{c} \rho \left(aa^{\prime }a,a\right)=\rho \left(aa^{*}a,a\right)=t=\rho \left(aa^{*},aa^{\prime }\right)=\rho \left(a^{\prime }a,a^{*}a\right). \end{array}$$

This yields that *ρ*(*aa*′aa*, aa*) ≥ *ρ*(*aa*′*a*, *a*) = *t* whence *ρ*(*aa*′aa*, aa*) = *t*. Thus,

(15) ρ(aa∗,a)≤ρ(aa′aa∗,aa′a)=ρ(aa′a,aa′aa∗)∧ρ(aa′aa∗,aa∗)≤ρ(aa′a,aa∗)∧ρ(aa∗,aa′)≤ρ(aa′a,aa′)=ρ(aa′,aa′a)∧ρ(aa′a,a)≤ρ(aa′,a).

By dual arguments, we can obtain that *ρ*(*aa*′, *a*) = *ρ*(aa*, *a*).

(2) In view of the fact that *a*′*b*(*a*′*b*)′, *aa*′ ∈ *E*(*S*), by Proposition 7, we have

(16) ρ(a′b(a′b)′,a′b)≤ρ(aa′b(a′b)′,aa′b)=ρ(a,aa′b)=ρ(a,b)=ρ(aa′b(a′b)′,b)≤ρ(a′aa′b(a′b)′,a′b)=ρ(a′b(a′b)′,a′b).

Thus, *ρ*(*a*′*b*(*a*′*b*)′, *a*′*b*) = *ρ*(*a*, *b*).

## 4. Normal Fuzzy Subsemigroups

In this section, we consider some basic properties of normal fuzzy subsemigroups of *E*-inverse semigroups.

**Definition 11**

Let *t* ∈ [0,1]. A fuzzy subset *μ* in *S* is called a* normal  fuzzy  subsemigroup  with  tip  t* in *S* if

1. (∀*x*, *y* ∈ *S*)  *μ*(*xy*) ≥ *μ*(*x*)∧*μ*(*y*),
2. (∀*a*, *x*, *y* ∈ *S*)  *t* ≥ *μ*(*a*) ≥ *μ*(*xay*)∧*μ*(*xy*),
3. (∀*e* ∈ *E*(*S*))  *μ*(*e*) = *t*.

We denote the set of normal fuzzy subsemigroups with tip *t* in *S* by **N** **F** **S**_*t*_(*S*) and let **N** **F** **S**(*S*) = ⋃_*t*∈[0,1]_ **N** **F** **S**_*t*_(*S*).

**Remark 12**

In fact, normal fuzzy subsemigroups with tip 1 are introduced in Zhang [11] where this class of fuzzy subsemigroups is called complete inner-unitary subsemigroups.

Let *G* be a group with identity *e*, *t* ∈ [0,1] and let *μ* be a fuzzy set in *G*. From Ajmal and Thomas [8], *μ* is called a* normal fuzzy subgroup* of *G* with tip *t* if for all *x*, *y* ∈ *G* the following conditions hold:

(17) $$\begin{array}{c} \mu \left(xy\right)\geq \mu \left(x\right)\wedge \mu \left(y\right),\;\;\mu \left(x^{-1}\right)=\mu \left(x\right),\\ \mu \left(xy\right)=\mu \left(yx\right),\;\;\mu \left(e\right)=t. \end{array}$$

We assert that normal fuzzy subsemigroups with tip *t* are generalizations of normal fuzzy subgroups with tip *t* in the range of *E*-inversive semigroups. To see this, we need the following result.

**Proposition**

Let *μ* ∈ **N** **F** **S**_*t*_(*S*), *a*, *b* ∈ *S*, and *a*′ ∈ *W*(*a*).

1. *μ*(*a*) = *μ*(*a*′).
2. *μ*(*a*) = *μ*(*aa*′*a*).
3. *μ*(*ab*) = *μ*(*ba*).

**Proof**

(1) On the one hand, we have *aa*′ ∈ *E*(*S*) and

(18) μ(a)≥μ(a′aa′)∧μ((a′)2)=μ(a′)∧μ((a′)2)=μ(a′).

On the other hand, since *a*′*a* ∈ *E*(*S*), we have *μ*(*a*′*a*) = *t* ≥ *μ*(*a*). This implies that

(19) μ(a′)≥μ(aa′a)∧μ(a2)≥(μ(a)∧μ(a′a))∧μ(a2)=μ(a).

Therefore, *μ*(*a*) = *μ*(*a*′).

(2) This follows from item (1) and the fact that *aa*′*a* ∈ *W*(*a*′).

(3) The result follows from Proposition 2.6 in Zhang [11].

The following result justifies the name of normal fuzzy subsemigroups.

**Theorem 14**

Let *G* be a group with identity *e*, *t* ∈ [0,1] and let *μ* be a fuzzy subset in *G*. Then *μ* is a normal fuzzy subsemigroup with tip *t* of *G* if and only if *μ* is a normal fuzzy subgroup of *G* with tip *t*.

**Proof**

Observe that *e* is the unique idempotent in *G* and the inverse *a* ^−1^ of *a* is certainly the unique weak inverse of *a* for all *a* ∈ *G*. If *μ* is a normal fuzzy subsemigroup with tip *t* of *G*, then by Proposition 13, *μ* is a normal fuzzy subgroup of *G* with tip *t*. Conversely, let *μ* be a normal fuzzy subgroup of *G* with tip *t* and *a*, *x*, *y* ∈ *S*. Then

(20) μ(a)=μ(x−1xayy−1)=μ(xayy−1x−1)≥μ(xay)∧μ(y−1x−1)

by condition (17). This implies that

(21) μ(a)≥μ(xay)∧μ(y−1x−1)=μ(xay)∧μ((xy)−1)=μ(xay)∧μ(xy).

Moreover, we have

(22) t=μ(e)=μ(aa−1)≥μ(a)∧μ(a−1)=μ(a)∧μ(a)=μ(a).

Thus, *μ* is a normal fuzzy subsemigroup of *G* with tip *t*.

Since ⋂_*i*∈*I*_ *σ*_*i*_ ∈ **N** **F** **S**_*t*_(*S*) for *σ*_*i*_ ∈ **N** **F** **S**_*t*_(*S*), *i* ∈ *I*, and the element

(23) $$\begin{array}{c} S⟶\left[0,1\right],\;\;a⟼t \end{array}$$

is the greatest one in **N** **F** **S**_*t*_(*S*), we have the following theorem by Lemma 2.

**Theorem 15**

(**N** **F** **S**_*t*_(*S*), ⊆) forms a lattice.

On normal fuzzy subsemigroups with tip *t* of *E*-inverse semigroups, we also have the following basic properties which will be used in the final section.

**Proposition**

Let *μ* ∈ **N** **F** **S**_*t*_(*S*), *e*, *f* ∈ *E*(*S*), *a*, *b* ∈ *S*, and *a*′, a* ∈ *W*(*a*), *b*′ ∈ *W*(*b*).

1. *μ*(*ef*) = *t*.
2. *μ*(*a*′*b*) = *μ*(*b*′*a*).
3. *μ*(*a*′*b*) = *μ*(a*b).

**Proof**

(1) Since *e*, *f* ∈ *E*(*S*), we have *μ*(*e*) = *μ*(*f*) = *t*. This implies that *μ*(*ef*) ≥ *μ*(*e*)∧*μ*(*f*) = *t* whence *μ*(*ef*) = *t*.

(2) The result follows from the facts that
  (24) μ(a′b)≥μ(aa′bb′)∧μ(ab′)=t∧μ(ab′)=μ(ab′)=μ(b′a),μ(b′a)=μ(ab′)≥μ(a′ab′b)∧μ(a′b)=t∧μ(a′b)=μ(a′b).
(3) This follows from the proof of Theorem 2.12 in Zhang [11].

## 5. The Relationship of **G** **F** **C**_*t*_(*S*) and **N** **F** **S**_*t*_(*S*)

In this section, we show that **G** **F** **C**_*t*_(*S*) is isomorphic to **N** **F** **S**_*t*_(*S*) as lattices whence **N** **F** **S**_*t*_(*S*) is modular for all *t* in [0,1]. We first give some useful propositions.

**Proposition 17**

Let *μ* ∈ **N** **F** **S**_*t*_(*S*) and

(25) $$\begin{array}{c} \rho_{\mu }:S\times S⟶\left[0,1\right],\;\;\left(a,b\right)⟼\mu \left(a^{\prime }b\right). \end{array}$$

Then *ρ*_*μ*_ ∈ **G** **F** **C**_*t*_(*S*), where *a*′ ∈ *W*(*a*).

**Proof**

In view of Proposition 16(3), the above *ρ*_*μ*_ is well defined. Now, let *a*, *b*, *c* ∈ *S* and *a*′ ∈ *W*(*a*), *b*′ ∈ *W*(*b*), *c*′ ∈ *W*(*c*). Then we have the following facts:

(1) Since *a*′*a* ∈ *E*(*S*), *ρ*_*μ*_(*a*, *a*) = *μ*(*a*′*a*) = *t*.
(2) By Proposition 16(2), we have
  (26) $$\begin{array}{c} \rho_{\mu }\left(a,b\right)=\mu \left(a^{\prime }b\right)=\mu \left(b^{\prime }a\right)=\rho_{\mu }\left(b,a\right)\leq t. \end{array}$$

(3) Since *b*′*b* ∈ *E*(*S*), it follows that *μ*(*b*′*b*) = *t* and

(27) μ(a′b)≥μ(ca′bb′)∧μ(cb′)≥μ(ca′)∧μ(bb′)∧μ(cb′)=μ(ca′)∧μ(cb′)=μ(a′c)∧μ(b′c)=μ(a′c)∧μ(c′b)

by Proposition 13(3) and Proposition 16(2). This implies that

(28) $$\begin{array}{c} \rho_{\mu }\left(a,b\right)\geq \rho_{\mu }\left(a,c\right)\wedge \rho_{\mu }\left(c,b\right). \end{array}$$

(4) For any (*ac*)′ ∈ *W*(*ac*), we have *ac*(*ac*)′ ∈ *E*(*S*) and

(29) μ((ac)′bc)≥μ(ac(ac)′bcc′b′)∧μ(acc′b′)≥μ(ac(ac)′)∧μ(bcc′b′)∧μ(acc′b′)=t∧μ(cc′b′b)∧μ(cc′b′a)=μ(cc′b′a)≥μ(cc′)∧μ(b′a)=t∧μ(b′a)=μ(b′a)=μ(a′b)

by Proposition 13(3) and Proposition 16(1), (2). This implies that *μ*(*a*′*b*) ≤ *μ*((*ac*)′*bc*). Dually, we have *μ*(*a*′*b*) ≤ *μ*((*ca*)′*cb*). This implies that

(30) $$\begin{array}{c} \mu \left(a^{\prime }b\right)\leq \mu \left({\left(ac\right)}^{\prime }bc\right)\wedge \mu \left({\left(ca\right)}^{\prime }cb\right). \end{array}$$

Thus, *ρ*_*μ*_(*a*, *b*) ≤ *ρ*_*μ*_(*ac*, *bc*)∧*ρ*_*μ*_(*ca*, *cb*).

(5) By Proposition 16 and the fact that *e* ∈ *W*(*e*), we have *ρ*_*μ*_(*e*, *f*) = *μ*(*ef*) = *t*.
(6) *ρ*_*μ*_(*a*, *aa*′*a*) = *μ*(*a*′*aa*′*a*) = *μ*(*aa*′) = *t*.

From the above six items, we can see that *ρ*_*μ*_ ∈ **G** **F** **C**_*t*_(*S*) by Proposition 6.

**Proposition 18**

Let *ρ* ∈ **G** **F** **C**_*t*_(*S*) and define

(31) $$\begin{array}{c} \mu_{\rho }:S⟶\left[0,1\right]:a⟼\rho \left(aa^{\prime },a\right). \end{array}$$

Then *μ*_*ρ*_ ∈ **N** **F** **S**_*t*_(*S*), where *a*′ ∈ *W*(*a*).

**Proof**

By Proposition 10(1), *μ*_*ρ*_ is well defined. Now, let

(32) $$\begin{array}{c} x,a,y\in S,\;x^{\prime }\in W\left(x\right),\;y^{\prime }\in W\left(y\right),\\ {\left(xy\right)}^{\prime }\in W\left(xy\right),\;{\left(xay\right)}^{\prime }\in W\left(xay\right). \end{array}$$

Then

(33) $$\begin{array}{c} x^{\prime }x,y^{\prime }y,xx^{\prime },yy^{\prime },\left(xy\right){\left(xy\right)}^{\prime }\in E\left(S\right). \end{array}$$

By Proposition 7, we have

(34) ρ((xy)(xy)′,xy)=ρ(yy′(xy)(xy)′,xy)=ρ(xx′yy′(xy)(xy)′,xy)≥ρ(xx′,x)∧ρ(yy′(xy)(xy)′,y)=ρ(xx′,x)∧ρ(yy′,y).

This implies that *μ*_*ρ*_(*xy*) ≥ *μ*_*ρ*_(*x*)∧*μ*_*ρ*_(*y*). On the other hand, also by Proposition 7, we have

(35) ρ(xay(xay)′,xay)∧ρ(xy(xy)′,xy) =ρ(xay,xay(xay)′xy(xy)′) ∧ρ(xay(xay)′xy(xy)′,xy) ≤ρ(xay,xy).

Observe that

(36)   ρ(xay,xy)≤ρ(x′xayy′,x′xyy′)=ρ(x′xayy′,x′xyy′aa′)=ρ(a,aa′)=ρ(aa′,a)

by Proposition 7. Thus, *t* ≥ *ρ*(*aa*′, *a*) = *μ*_*ρ*_(*a*) ≥ *μ*_*ρ*_(*xay*)∧*μ*_*ρ*_(*xy*). Finally, since *e* ∈ *W*(*e*) for all *e* ∈ *E*(*S*), we have *μ*_*ρ*_(*e*) = *ρ*(*ee*, *e*) = *ρ*(*e*, *e*) = *t* for all *e* ∈ *E*(*S*). Therefore, *μ*_*ρ*_ ∈ **N** **F** **S**_*t*_(*S*).

At this stage, we can give the main result of this paper.

**Theorem 19**

The mappings

(37) $$\begin{array}{c} \Psi_{t}:GFC_{t}\left(S\right)⟶NFS_{t}\left(S\right),\;\;\rho ⟼\mu_{\rho };\\ \Phi_{t}:NFS_{t}\left(S\right)⟶GFC_{t}\left(S\right),\;\;\mu ⟼\rho_{\mu } \end{array}$$

are mutually inverse bijections preserving the inclusion relations, where *μ*_*ρ*_ and *ρ*_*μ*_ are defined as in Propositions 17 and 18, respectively.

**Proof**

From Propositions 17 and 18, the above mappings are well defined. Now, let *μ* ∈ **N** **F** **S**_*t*_(*S*), *a* ∈ *S*, and *a*′ ∈ *W*(*a*). Then *aa*′ ∈ *W*(*aa*′). This implies that

(38) ΨtΦt(μ)(a)=Ψt(ρμ)(a)=ρμ(aa′,a)=μ(aa′a)=μ(a)

by Proposition 13(2). On the other hand, for any *a*, *b* ∈ *S* and *a*′ ∈ *W*(*a*), (*a*′*b*)′ ∈ *W*(*a*′*b*), we have

(39) ΦtΨt(ρ)(a,b)=Φt(μρ)(a,b)=μρ(a′b)=ρ(a′b(a′b)′,a′b)=ρ(a,b)

by Proposition 10(2). This implies that Ψ_*t*_ and Φ_*t*_ are mutually inverse bijections. Obviously, Ψ_*t*_ and Φ_*t*_ preserve the inclusion relations.

**Corollary 20**

The lattices (**G** **F** **C**_*t*_(*S*), ⊆) and (**N** **F** **S**_*t*_(*S*), ⊆) are isomorphic. As a consequence, the lattice (**N** **F** **S**_*t*_(*S*), ⊆) is also modular.

**Proof**

By Lemma 1 and Theorem 19, the lattice (**G** **F** **C**_*t*_(*S*), ⊆) is isomorphic to the lattice (**N** **F** **S**_*t*_(*S*), ⊆). Thus, the lattice (**N** **F** **S**_*t*_(*S*), ≤) is also modular by Theorem 9.

**Corollary 21**

(**G** **F** **C**(*S*), ⊆) and (**N** **F** **S**(*S*), ⊆) are two mutually isomorphic lattices.

**Proof**

It is routine to check that

(40) $$\begin{array}{c} \left(\underset{i\in I}{⋂}\rho_{i}\right)\in GFC_{\wedge_{i\in I}t_{i}}\left(S\right),\;\;\left(\underset{j\in J}{⋂}\sigma_{j}\right)\in NFS_{\wedge_{j\in J}t_{j}}\left(S\right) \end{array}$$

for *ρ*_*i*_ ∈ **G** **F** **C**_*t*_*i*__(*S*), *i* ∈ *I*, and *σ*_*j*_ ∈ **N** **F** **S**_*t*_*j*__(*S*), *j* ∈ *J*, where *I* and *J* are index sets. Moreover,

(41) $$\begin{array}{c} \rho_{1}:S\times S⟶\left[0,1\right],\;\;\left(a,b\right)⟼1,\\ \sigma_{1}:S⟶\left[0,1\right],\;\;x⟼1 \end{array}$$

are the greatest elements in (**G** **F** **C**(*S*), ⊆) and (**N** **F** **S**(*S*), ⊆), respectively. By Lemma 2, (**G** **F** **C**(*S*), ⊆) and (**N** **F** **S**(*S*), ⊆) are two lattices. Moreover, if we let

(42) $$\begin{array}{c} \Psi :GFC\left(S\right)⟶NFS\left(S\right),\;\rho ⟼\Psi_{t}\left(\rho \right),\;\rho \in GFC_{t}\left(S\right),\\ \Phi :NFS\left(S\right)⟶GFC\left(S\right),\;\rho ⟼\Phi_{t}\left(\sigma \right),\;\sigma \in NFS_{t}\left(S\right), \end{array}$$

then by Theorem 19, Ψ and Φ are mutually inverse bijections which preserve the inclusion relations and thus (**G** **F** **C**(*S*), ⊆) and (**N** **F** **S**(*S*), ⊆) are isomorphic from Lemma 1.

We end this section by giving an example to illustrate our previous results.

**Example 22**

Let *S* be a semigroup with the following multiplication table:

(43) Saefbaeaaeeaeeafaefabeabe

Then *S* is an *E*-inversive semigroup which is nonregular. Moreover,

(44) $$\begin{array}{c} E\left(S\right)=\left\{e,f\right\},\;\;W\left(a\right)=\left\{a\right\},\;\;W\left(e\right)=\left\{e,f\right\},\\ W\left(f\right)=\left\{f\right\},\;\;W\left(b\right)=\left\{a\right\}. \end{array}$$

Fix an element *t* in the interval [0,1]. For every *s* ∈ [0,1] with *s* ≤ *t*, define a fuzzy set *μ*_*s*_ in *S* as follows:

(45) $$\begin{array}{c} \mu_{s}:S\to \left[0,1\right],\;e⟼t,\;f⟼t,\;a⟼s,\;b⟼s. \end{array}$$

It is routine to check that *μ*_*s*_ is a normal fuzzy subsemigroup with tip *t* in *S*. Furthermore, in view of the fact that

(46) $$\begin{array}{c} E\left(S\right)=\left\{e,f\right\},\;\;W\left(a\right)=\left\{a\right\},\;\;W\left(e\right)=\left\{e,f\right\},\\ W\left(f\right)=\left\{f\right\},\;\;W\left(b\right)=\left\{a\right\} \end{array}$$

and Proposition 13, we have

(47) $$\begin{array}{c} NFS_{t}\left(S\right)=\left\{\mu_{s}\;|\;s\leq t,s\in \left[0,1\right]\right\}. \end{array}$$

Let *μ*_*s*_ ∈ **N** **F** **S**_*t*_(*S*). By Proposition 17, we can define

(48) $$\begin{array}{c} \rho_{\mu_{s}}:S\times S⟶\left[0,1\right],\;\left(x,y\right)⟼\mu \left(x^{\prime }y\right),\;x^{\prime }\in W\left(x\right). \end{array}$$

More precisely, *ρ*_*μ*_*s*__ satisfies that

(49) ρμs(a,a)=ρμs(e,e)=ρμs(f,f)=ρμs(b,b)=ρμs(a,b)=ρμs(b,a)=ρμs(e,f)=ρμs(f,e)=t,ρμs(a,e)=ρμs(e,a)=ρμs(f,a)=ρμs(a,f)=ρμs(e,b)=ρμs(b,e)=ρμs(b,f)=ρμs(f,b)=s.

Then *ρ*_*μ*_*s*__ ∈ **G** **F** **C**_*t*_(*S*). By Theorem 19, **G** **F** **C**_*t*_(*S*) = {*ρ*_*μ*_*s*__ | *s* ≤ *t*, *s* ∈ [0,1]}. By virtue of Corollary 20, (**G** **F** **C**_*t*_(*S*), ⊆) and (**N** **F** **S**_*t*_(*S*), ⊆) are isomorphically modular lattices.

## 6. Conclusion

In this paper, we have introduced and investigated the lattices of group fuzzy congruences and normal fuzzy subsemigroups on *E*-inversive semigroups. Our results generalize the corresponding results of groups and regular semigroups. From the results presented in the paper, the lattices of group fuzzy congruences and normal fuzzy subsemigroups on *E*-inversive semigroups can be regarded as a source of possibly new modular lattices. On the other hand, this paper also leaves some questions which can be considered as future works. For example, from Ajmal and Thomas [8], if *S* is a group, then (**N** **F** **S**(*S*), ⊆) is a modular lattice. Thus, the following question would be interesting: is (**N** **F** **S**(*S*), ⊆) also modular for an *E*-inversive semigroup *S*?
